# Determinants of the Sympatric Host-Pathogen Relationship in Tuberculosis

**DOI:** 10.1371/journal.pone.0140625

**Published:** 2015-11-03

**Authors:** Susana David, A. R. A. Mateus, Elsa L. Duarte, José Albuquerque, Clara Portugal, Luísa Sancho, João Lavinha, Guilherme Gonçalves

**Affiliations:** 1 Departamento de Genética Humana, Instituto Nacional de Saúde Doutor Ricardo Jorge (INSA), Lisboa, Portugal; 2 Instituto Gulbenkian de Ciência (IGC), Oeiras, Portugal; 3 Institute of Biology, Leiden University, Leiden, The Netherlands; 4 Escola de Ciências e Tecnologia/ Instituto de Ciências Agrárias e Ambientais Mediterrânicas (ICAAM), Universidade de Évora, Évora, Portugal; 5 Serviço de Patologia Clínica, Hospital Fernando Fonseca, Amadora, Portugal; 6 Unidade Multidisciplinar de Investigação Biomédica (UMIB), Instituto de Ciências Biomédicas Abel Salazar (ICBAS), Porto, Portugal; University of Padova, Medical School, ITALY

## Abstract

Major contributions from pathogen genome analysis and host genetics have equated the possibility of *Mycobacterium tuberculosis* co-evolution with its human host leading to more stable sympatric host–pathogen relationships. However, the attribution to either sympatric or allopatric categories depends on the resolution or grain of genotypic characterization. We explored the influence on the sympatric host-pathogen relationship of clinical (HIV infection and multidrug-resistant tuberculosis [MDRTB]) and demographic (gender and age) factors in regards to the genotypic grain by using spacer oligonucleotide typing (spoligotyping) for classification of *M*. *tuberculosis* strains within the Euro-American lineage. We analyzed a total of 547 tuberculosis (TB) cases, from six year consecutive sampling in a setting with high TB-HIV coinfection (32.0%). Of these, 62.0% were caused by major circulating pathogen genotypes. The sympatric relationship was defined according to spoligotype in comparison to the international spoligotype database SpolDB4. While no significant association with Euro-American lineage was observed with any of the factors analyzed, increasing the resolution with spoligotyping evidenced a significant association of MDRTB with sympatric strains, regardless of the HIV status. Furthermore, distribution curves of the prevalence of sympatric and allopatric TB in relation to patients’ age showed an accentuation of the relevance of the age of onset in the allopatric relationship, as reflected in the trimodal distribution. On the contrary, sympatric TB was characterized by the tendency towards a typical (standard) distribution curve. Our results suggest that within the Euro-American lineage a greater degree of genotyping fine-tuning is necessary in modeling the biological processes behind the host-pathogen interplay. Furthermore, prevalence distribution of sympatric TB to age was suggestive of host genetic determinisms driven by more common variants.

## Introduction

Tuberculosis (TB) first became an epidemic disease in the crowded urban conditions of the industrial revolution. During the 18^th^ to 19^th^ century, the disease caused 20% of all deaths in Europe in an unparalleled epidemic, setting the stage for the long-term presence and global expansion of TB. In 1958, from the analysis of morbidity and mortality data, E. R. N. Grigg concluded, that a TB epidemic lasts approximately 300 years [1–2]. Subsequent studies suggested that the reduced susceptibility of Europeans to pulmonary TB today would unlikely be due to the TB epidemic alone [3]. Nevertheless, based on Griggs assumptions, the low level of TB disease currently observed would be suggestive of its final decline. In this scenario, one would expect, as commonly observed on national European scales, a steady rise of the age of the at-risk group, a marker of the epidemic evolution of the disease [4].

Recently, pathogen genetics has been able to substantiate earlier findings recognizing the geographic distribution of *Mycobacterium tuberculosis*. Observations, dating back to the 1970’s, reported the geographic confinement of TB caused by *M*. *africanum* to certain regions in the African continent in spite of the historical mobility of these populations during the transatlantic slave trade from the 16^th^ through to the 19^th^ centuries [5]. It was not until the 1990s, that the molecular tools were available to bring the necessary insight into the evolutionary epidemiology of TB and the acknowledgment of *M*. *tuberculosis* phylogeographical lineages. These were defined through a whole genome approach using Large Sequence Polymorphism (LSP) analysis on a sample from the San Francisco metropolitan area. They included the Indo-Oceanic, East-Asian, East-African-Indian, Euro-American, West African 1 and West African 2 lineages [6–7]. Reflecting the clonal genetic population structure of *M*. *tuberculosis*, spacer oligonucleotide typing (spoligotyping) based on deletion analysis within a single genomic locus, the Direct Repeat (DR), has defined spoligotype families congruent to the LPS defined lineages [8–9]. The DR is a clustered regularly interspaced short palindromic repeats locus (CRISPR). CRISPR are gaining relevance in the evolutionary reconstruction of bacterial pathogens including of *M*. *tuberculosis* [10]. Although spoligotyping is not a genome wide analysis and may be subject to convergent evolution [11–12], it is considered a phylogenetically informative tool [8–10]. A global analysis of spoligotype patterns from thousands of strains [13–15], *M*. *tuberculosis* spoligotype families include the East-African-Indian (EAI), Beijing, Central-Asian (CAS) and *M*. *africanum* congruent with the LSP defined lineages Indo-Oceanic, East-Asian, East-African-Indian and the West African (West African 1 and West African 2), respectively. Also, the spoligotype families Latin-American-Mediterranean (LAM), Haarlem (H), T, S and X, together, account for the LSP Euro-American lineage. These and other genotyping tools have proven useful to assess *M*. *tuberculosis* strain geographic distribution in accordance to recent and ancient human migrations [14–21].

In Portugal, previous studies have delineated the geographic specificity of *M*. *tuberculosis* genotypes [22–25]. More specifically, using one isolate per TB patient from consecutively sampled 665 TB cases over a six year period, spoligotypes characteristic of Portuguese or Portuguese related settings were identified [23]. These genotypes were suspected to be linked to the Portuguese colonial period because of the similarities between *M*. *tuberculosis* population structures observed in Portugal and Brazil [23,25]. Historical ties between Portugal and Brazil are profound. In 1808, the capital of the Portuguese colonial empire was transferred from Lisbon first to Salvador (Bahia) and later to Rio de Janeiro, as a means for its protection during the Napoleonic invasion, rendering sociocultural ties between Portugal and Brazil at a peak during the great “White Plague” epidemic.

Due to major differences observed in the immune response to TB in naïve populations, it is considered to have exerted a powerful selective pressure on human evolution, helping to change the nature of human immune responses to infection [26–28]. Other than the differences in the host’s immunological response to infection, the influence of the infective strain on disease phenotype has been gaining attention. An increasing number of studies reported associations between human genetic variants and particular *M*. *tuberculosis* lineages [29–33]. Thus, these have been classified as sympatric host-pathogen relationships, when host and pathogen share a common ancestral geographic origin, or allopatric, when they originate from non-overlapping geographic areas [34]. Accordingly, the more stable associations between these lineages and their human populations would characterize the sympatric host-pathogen relationship [6–7,35]. Although useful in helping to model divergence, attribution of host-pathogen relationships to one of these two categories depends on the resolution of strain genotypic characterization, having subsequent implications on the understanding of divergence and the modeling of genetic events [36]. However, in spite of these many contributions, “co-evolution” of TB with its human host is a model at the dawn of its understanding.

The occurrence of immune-compromising diseases is likely to influence the host-pathogen relationship. In a recent molecular-epidemiological study, Fenner and collaborators identified human immunodeficiency virus (HIV) infection as a possible disruptor of the relationship of locally adapted *M*. *tuberculosis* strains to the host [37]. They considered the combination of a particular strain lineage and its corresponding patient population as sympatric (e.g. Euro-American lineage in Europeans) or allopatric (e.g. East-Asian lineage in Europeans) and concluded that HIV infection was associated with the less adapted allopatric lineages among patients born in Europe, providing evidence that the sympatric host–pathogen relationship in TB was disrupted by HIV.

Here, we reanalyze our current understanding of the *M*. *tuberculosis* population structure in Portugal and its particular phylogeographic characteristics. Accordingly, using a retrospective molecular epidemiology approach we investigated the influence of HIV infection and other clinical and demographic factors on the sympatric host-pathogen relationship. Finally, we discussed our findings in terms of their possible impact on future investigations on the TB host-pathogen relationship.

## Materials and Methods

### Patient characteristics

This retrospective molecular epidemiology study included 859 TB patients with positive culture for *M*. *tuberculosis*, having been diagnosed and treated according to recommended procedures in Portugal, between the years 1999 and 2006. The corresponding *M*. *tuberculosis* isolates included 665 isolates obtained between 1999 and 2005 through consecutive sampling of one isolate per TB patient hospitalized in the Greater Lisbon area [23], and a convenience sample from 2006 of 69 isolates from the Lisbon district, 74 from the Porto district and 51 from various locations within Portugal. Socio-demographic data (age, sex) and laboratory parameters (*M*. *tuberculosis* drug susceptibility status to first line drugs, HIV infection status) were obtained from the hospital fully anonymized and associated only with the *M*. *tuberculosis* isolate number.

A total of 547 TB patients with known HIV status, 82.3% (547/665) of the total number of cases, were included in the study. Of these 32.0% (175/547) were HIV–infected and 68.0% (372/547) HIV–negative. Conventional susceptibility testing for first line anti-TB drugs (streptomycin, isoniazid, rifampicin, ethambutol) was carried out using the Bactec 960 TB system (Becton Dickinson, Quilaban, Portugal) and was available for 86.7% (474/547) of the cases.

Since important spoligotype variability was observed [23], to avoid artifacts, a “major subgroup” of 339 cases was defined as those cases in which the *M*. *tuberculosis* shared international types (SITs) grouped 15 or more isolates, representing 62.0% (339/547) of the patients included in the study. This arbitrary cutoff for the number of isolates was applied to the entire 859 TB patient dataset and not to the 547 subgroup of patients with known HIV status, in order to avoid any bias that may have occurred in the prescription of HIV diagnosis directed towards risk groups. Drug susceptibility status was available for 86.7% (294/339) of these cases.

### Molecular analysis

The genotyping of the 859 *M*. *tuberculosis* isolates in this study was performed by spoligotyping using the Spoligotyping Kit according to the manufacturer’s instructions (Isogen Bioscience BV, Maarsen, Netherlands) and as previously described [23,25]. SIT enumeration was in agreement to the international spoligotype database SpolDB4 (http://www.pasteur-guadeloupe.fr:8081/SITVITdemo) [15]. Spoligotype patterns were classified into spoligotype families using the SPOTCLUST program (http://cgi2.cs.rpi.edu/-bennek/SPOTCLUST.html) [38].

All the spoligotype patterns were subject to phylogenetic analysis using the MIRU-VNTR-plus database (http://www.MIRU-VNTRplus.org) [39–40]. A minimum spanning tree was calculated by the program according to Kruskal’s algorithm and a force-directed graph layout used for visualization. MIRU-VNTRplus spoligotype derived clonal complexes (CC) and Singletons (not grouped) were obtained through single locus variation (SLV), reflecting a single spoligotyping spacer difference.

### Sympatric versus allopatric classification of strain genotypes

Consecutive sampling of one isolate per patient from 1999 to 2005 was used in attributing local specificity of SITs [23]. A dataset of 665 SITs was generated representing all the TB cases diagnosed during this time period at the Fernando Fonseca Hospital, which deserves a densely populated area of greater Lisbon. From the analysis of this dataset, SITs were considered characteristic of Portuguese or Portuguese related settings when their relative frequency weighed heavily in that observed in the European countries of traditional Portuguese immigration (Belgium, France, Germany, Great Britain, Ireland, Luxembourg, Netherlands, Spain and Switzerland), with over 60% of the isolates, or the SpolDB4 as a whole, with over 10% of the isolates [23]. The Portuguese related SITs all belonged to the LAM and T spoligotype families, included in the Euro-American lineage [8–9]. In this study, following Fenner and collaborators proposal [37], strains with these highly prevalent locally adapted genotypes were referred to as sympatric. Historically Portugal was not a country of immigration. The major wave was relative to the decolonization period in the 1970s. Today, these populations constitute Portuguese born second and third generations. Therefore, in our setting, place of birth was not relevant as a proxy of the ancestry of the study population as in other settings [6–7,35,37]. These included the LAM1 sub-family SIT20, where the Portuguese subset alone accounted for 21% of the worldwide representativeness of the genotype, the T1 sub-family SIT244 accounting for 53%, the LAM6 sub-family SIT64 accounting for 11%, the LAM1 sub-family SIT389 accounting for 68% and the LAM9 sub-family SIT 1106 accounting for 83%.

The distribution amongst sympatric and allopatric strains was also analyzed within the “major subgroup” of 339 cases. Accordingly, five genotypes from the “major subgroup” were attributed to the sympatric group of strains (SIT20, SIT244, SIT64, SIT389, and SIT 1106). These spoligotypes represented 46.5% (229/493) of the major circulating genotypes in this study whereas the remaining spoligotypes considered as allopatric (SIT42, SIT150, SIT17, SIT53, SIT50, SIT1, SIT34 and SIT47) represented 53.5% (264/493).

### Influence of the infectious strain on patient age distribution

We analyzed the distribution of TB caused by particular pathogen genotypes, looking at the frequency of cases as a function of the age group categories in years: 0 to 4, 5 to 14, 15 to 24, 25 to 34, 35 to 44, 45 to 54, 55 to 64, and over 65. As HIV infection is a more recent epidemic and associated to specific usually younger risk groups in the Portuguese population [39], in order not to warp observations regarding evolutionary tendencies of the sympatric relationship between *M*. *tuberculosis* and its human host, these curves were analyzed in HIV-negative individuals considered as the reference group. Therefore, the typical trimodal distribution curves of incidence to age group were verified for genotypic groupings of *M*. *tuberculosis* isolates in HIV negative individuals in order to further qualify the sympatric compared to allopatric host–pathogen combinations.

### Statistical analysis

For descriptive statistics, the binary variables were expressed as percentages and the continuous variables as means.

All data analysis was performed with R (R Development Core Team 2005).

We tested the association of *M*. *tuberculosis* Euro-American lineage, considered by previous authors to be in a sympatric relationship with Europeans, relative to HIV infection (two levels: infected or negative), MDRTB (two levels: presence or absence), gender (two levels: male, female) and age (eight levels: 1: 0 to 4 years; 2: 5 to 14 years; 3: 15 to 24 years; 4: 25 to 34 years; 5: 35 to 44 years; 6: 45 to 54 years; 7: 55 to 64 years; 8: over 65 years). For this purpose we used the generalized linear model *Tuberculosis with a Euro-American lineage strain ~ HIV infection + MDRTB + gender + age group* assuming a binomial distribution of the error. Differences for the different factors tested in the overall generalized linear model were considered significant when the p-value ≤ 0.05 (Anova). We then tested in relation to *MIRU-VNTR-plus* spoligotype derived clonal complexes, using the generalized linear model *Tuberculosis with strains from MIRU-VNTR-plus Clonal Complexes ~ HIV infection + MDRTB + gender + age group* assuming a binomial distribution of the error as in the above. Finally, for our final model we tested in the same manner sympatric TB SITs, *Tuberculosis with a sympatric strain ~ HIV infection + MDRTB + gender + age group*, for both HIV-infected and HIV-negative patients, combined, and for the HIV-negative patients alone. In order to avoid artifacts in our analysis resulting from rare genotypes, the model was tested for the entire dataset (n = 547) as well as for the more frequent genotypes included in the “major subgroup” (n = 339). Odds ratios were derived for our final models. The interactions between factors were tested but, because they were not statistically significant, they were not included in our final models.

We also tested the effect of age on TB prevalence using the generalized linear models *Tuberculosis prevalence ~ Sympatric tuberculosis + Age group*, using the HIV-negative patients as the reference group, assuming a Poisson distribution of the error, with sympatric TB (two levels: presence or absence) and age group (eight levels: 1: 0 to 4 years; 2: 5 to 14 years; 3: 15 to 24 years; 4: 25 to 34 years; 5: 35 to 44 years; 6: 45 to 54 years; 7: 55 to 64 years; 8: over 65 years). When significant differences were found for the different factors in the overall models (Anova, p-value ≤ 0.05) we performed post-hoc pairwise comparisons between factor levels using Tukey’s honest significant differences (HSD) tests (alpha = 0.01).

## Results

Molecular analysis of the infectious strains was obtained for a full set of 547 TB cases (S1 and S2 Tables). In this table, genotypes were listed according to spoligotype pattern (SIT, shared international type). Spoligotype families [38] as well as the Large Sequence Polymorphism (LSP) defined phylogeographic lineages [7] are shown for each SIT. The predominant lineage was the Euro-American lineage, 92.1% (504/547). The MIRU-VNTRplus program was used for additional subdivision of the Euro-American lineage into spoligotype derived clonal complexes [39–40].

**Fig 1:**
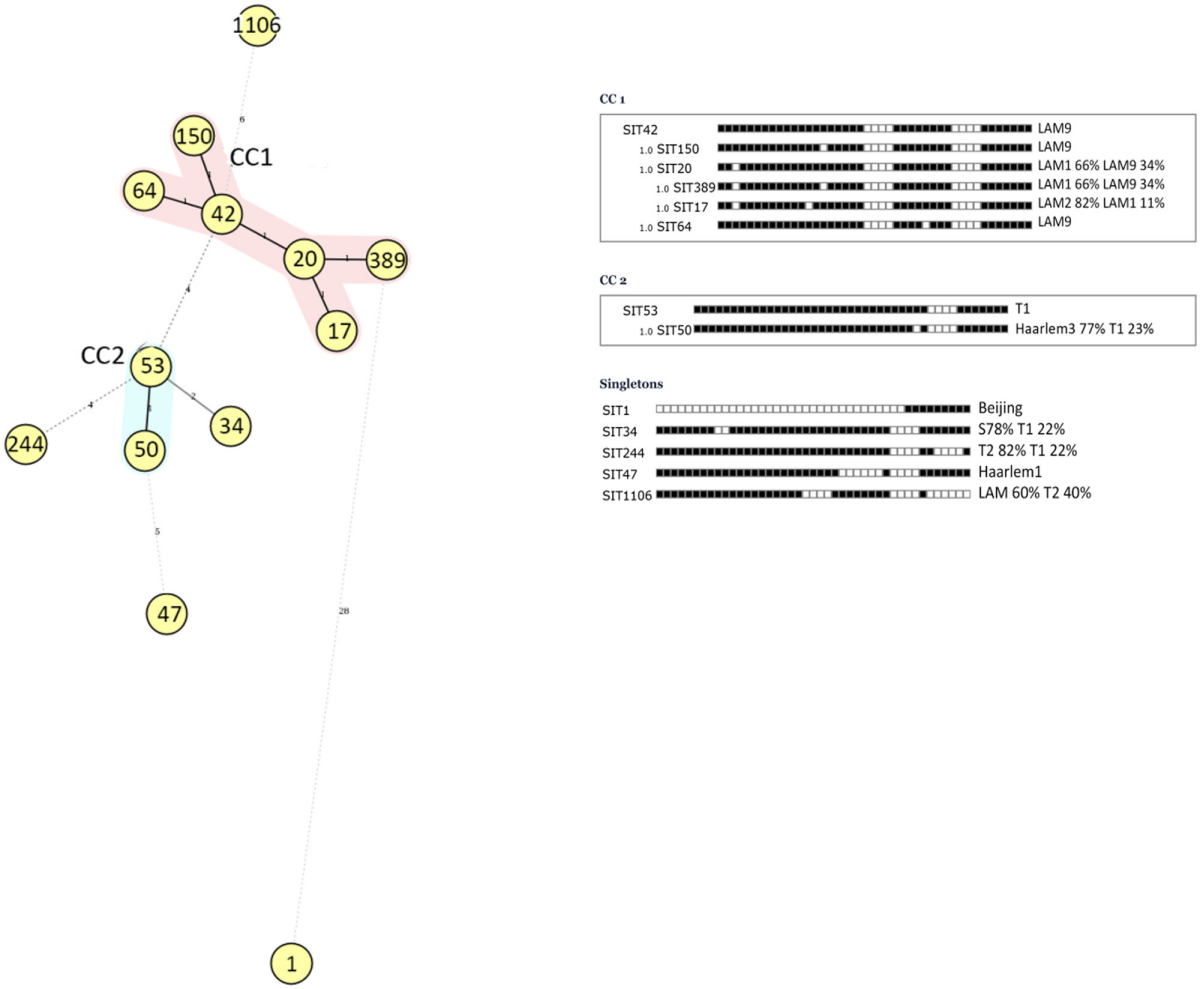
Phylogenetic analysis using the MIRU-VNTR-plus software (http://www.MIRU-VNTRplus.org) [39–40] for spoligotype patterns of the “major subgroup” of 339 cases, 62.0% (339/547), attributed to predominant *M*. *tuberculosis* genotypes (SITs). A minimum spanning tree was calculated according to Kruskal’s algorithm (Kruskal 1956) and a force-directed graph layout used for visualization. MIRU-VNTRplus clonal complexes (CC) were obtained through single locus variation (SLV), reflecting a single spoligotype spacer difference.

Descriptive analysis of demographic and clinical factors between the allopatric and sympatric strains is shown for the “major subgroup” (n = 339) in Table 1. HIV status was 33.3% (113/339) HIV–infected and 66.7% (226/339) HIV–negative. Age distribution is shown relative to the HIV-infected and HIV-negative groups. Furthermore, 56.3% (191/339) were infected with allopatric strains and 43.7% (148/339) with sympatric. In this “major subgroup”, all but the Beijing strains belong to Euro-American lineage, 95.6% (324/339). A phylogenetic tree representing the infectious strains was obtained by MIRU-VNTRplus software (Fig 1). The major spoligotype familly was the LAM, mainly grouped into the MIRU-VNTR PLUS spoligotype derived clonal complex CC1. As described in the methods section, the sympatric strains were represented by SITs 20, 64, 389, 244 and 1106 whereas the allopatric strains were represented by SITs 42, 150, 17, 53, 50, 1, 34, 47. The sympatric strains were all classified by the SPOTCLUST program as LAM strains except SIT244 (T2 82% T1 18%), included within the T family, and SIT1106 (LAM9 60% T2 40%). Corroborating the *M*. *tuberculosis* population structure of the six year consecutive sampling from TB cases in the greater Lisbon area [23], the sympatric strains, except SIT244, were all identified in the independent convenience samples from the Lisbon and Porto districts [41–42] (S3 and S4 Tables, respectively).

**Table 1:** Tuberculosis patient characteristics according to genotype for the more frequently observed *Mycobacterium tuberculosis* strain genotypes.

Strain genotype^1^							Patient characteristics					
	Spoligotype neighbor joining tree^2^						Male sex (%)	MDR^4^ (%)	HIV^5^ infection (%)	Age^6^ (mean ± sd)		
	CC	Node	SLV (1)	SLV (2)	Lineage (Spoligotype)^3^	Freqency (%)				All	HIV+	HIV-
All (n = 339)	-	-	-	-	-	100.0% (339/339)	72.0% (244/339)	9.9% (29/294)	33.3% (113/339)	40.42 ± 16.84	37.03 ± 10.21	42.12 ± 19.11
Allopatric (n = 191)	-	-	-	-	-	56.3% (191/339)	72.3% (138/191)	2.4% (4/164)	27.2% (52/191)	40.68 ± 17.05	37.96 ± 10.23	41.70 ± 18.92
Sympatric (n = 148)	-	-	-	-	-	43.7% (148/339)	71.6% (106/148)	19.2% (25/130)	41.2% (61/148)	40.09 ± 16.60	36.23 ± 10.21	42.79 ± 19.51
	CC1					59.0% (200/339)	75.0% (150/200)	11.5% (20/174)	33.0% (66/200)	42.00 ± 17.43	37.83 ± 10.62	44.04 ± 19.66
		SIT42			Euro-American (LAM9)	18.0% (61/339)	72.1% (44/61)	3.8% (2/52)	32.8% (20/61)	43.21 ± 16.90	38.70 ± 8.34	45.41 ± 19.50
			SIT150		Euro-American (LAM9)	6.0% (20/339)	85.0% (17/20)	0.0% (0/18)	30.0% (6/20)	40.50 ± 17.22	39.83 ± 16.61	40.79 ± 18.08
			SIT20		Euro-American (LAM1 66% LAM9 34%)	24.2% (82/339)	79.3% (65/82)	20.8% (15/72)	36.6% (30/82)	41.85 ± 18.45	38.07 ± 12.03	44.04 ± 21.09
				SIT17	Euro-American (LAM2 82% LAM1 11%)	2.9% (10/339)	50.0% (5/10)	0.0% (0/8)	30.0% (3/10)	38.80 ± 16.43	32.00 ± 3.46	41.71 ± 19.18
				SIT389	Euro-American (LAM1 66% LAM9 34%)	3.8% (13/339)	69.2% (9/13)	23.1% (3/13)	30.8% (4/13)	39.46 ± 9.20	38.50 ± 5.20	39.89 ± 10.78
			SIT64		Euro-American (LAM9)	4.1% (14/339)	71.4% (10/14)	0.0% (0/11)	21.4% (3/14)	44.29 ± 21.99	30.67 ± 4.04	48.00 ± 23.55
	CC2					15.3% (52/339)	71.2% (37/52)	4.7% (2/43)	26.9% (14/52)	39.48 ± 16.86	39.64 ± 11.58	39.42 ± 18.57
		SIT53			Euro-American (T1)	11.5% (39/339)	71.8% (28/39)	3.2% (1/31)	23.1% (9/39)	39.77 ± 16.80	38.22 ± 7.21	40.23 ± 18.82
			SIT50		Euro-American (H3 77% T1 23%)	3.8% (13/339)	69.2% (9/13)	8.3% (1/12)	38.5% (5/13)	38.62 ± 17.72	42.20 ± 17.87	36.38 ± 18.47
	Singletons					25.7% (87/339)	65.5% (57/87)	9.1% (7/77)	37.9% (33/87)	37.37 ± 15.06	34.30 ± 8.32	39.24 ± 17.80
		SIT244			Euro-American (T2 82% T1 18%)	8.8% (30/339)	56.7% (17/30)	7.1% (2/28)	63.33% (19/30)	34.63 ± 9.60	33.63 ± 7.95	36.36 ± 12.18
		SIT1			East-Asian (Beijing)	4.4% (15/339)	53.3% (8/15)	0.0% (0/12)	20.0% (3/15)	35.27 ± 18.51	31.33 ± 6.66	36.25 ± 20.56
		SIT34			Euro-American (S 78% T1 22%)	3.5% (12/339)	75.0% (9/12)	0.0% (0/11)	25.0% (3/12)	44.33 ± 25.45	33.00 ± 8.72	48.11 ± 28.41
		SIT1106			Euro-American (LAM9 60% T2 40%)	2.7% (9/339)	55.6% (5/9)	83.3% (5/6)	55.6% (5/9)	36.56 ± 13.63	36.60 ± 10.74	36.50 ± 18.48
		SIT47			Euro-American (Haarlem1)	6.2% (21/339)	85.7% (18/21)	0.0% (0/20)	14.3% (3/21)	39.14 ± 11.13	39.00 ± 11.00	39.17 ± 11.47

^1^ The Allopatric and Sympatric classification of *M*. *tuberculosis* strains was obtained from comparison of Portuguese spoligotypes with the fourth international spoligotyping database SpolDB4 [23]. This database classifies strains according to their ‘‘Shared international type” (SIT). Allopatric strains include strains with SITs 42, 150, 17, 53, 50, 1, 34, 47 (n = 191) and Sympatric strains include those strains with SITs 20, 64, 389, 244, 1106 (n = 148).

^2^ The MIRU-VNTR plus web application (http://www.miru-vntrplus.org) was used to analyse spoligotype data of the *M*. *tuberculosis* isolates [39–40]. For minimum spanning tree analysis the Kruskal’s algorithm and force-directed graph layout for visualization according to the SIT using one single locus variation from the Node, SLV(1) was used to generate groupings into clonal clusters (CC).

^3^ *M*. *tuberculosis* lineages were classified using spoligotype data. Spoligotype designation was obtained from the SpolDB4 database (http://www.pasteur-guadeloupe.fr:8081/SITVITdemo) [14–15] and using the Spotclust program (http://www.rpi.edu/$bennek/EpiResearch) [38]. Lineage designation, East-Asian and Euro-American, was on Long Sequence Polymorphism (LSP) analysis [7,9].

^4^ Drug susceptibility data was available for 86.7% (294/339) of the cases and was used to determine the percentage of multidrugresistant tuberculosis (MDRTB) cases to strain genotype.

^5^ HIV = human immunodeficiency virus infection

^6^ Age in years was expressed as the mean ± sd, sd = standard deviation.

Statistical analysis of strain classification according to genotype and various parameters (HIV infection, MDRTB, gender and age) performed for the full dataset (n = 547), showed no statistical significance in the association between these and the Euro-American lineage (Table 2). A statistically significant association with male gender for two clonal complexes derived from MIRU-VNTRplus analysis was observed, CC1 p = 0.24 and CC4 p = 0.03. Furthermore, an association with HIV co-infection was observed for the Singletons (p = 0.03) and CC4 (p < 0.001).

**Table 2:** Characteristics of tuberculosis patients (n = 547) by presence of Euro-American lineage or MIRU-VNTRplus spoligotype clonal complex (CC and Singletons) *M*. *tuberculosis* strains.

	overall generalized linear model^1^ significance (p-value)			
	MDRTB	Gender	HIV infection	Age group
Euro-American	NS	NS	NS	NS
CC1	NS	0.024 *	NS	NS
CC2	NS	NS	NS	NS
CC3	NS	NS	NS	NS
CC4	NS	0.031 *	<0.001 ***	NS
CC5	NS	NS	NS	NS
Other CC	NS	NS	NS	NS
Singletons	NS	NS	0.030 *	NS

^1^ The MIRU-VNTR plus web application (http://www.miru-vntrplus.org) was used to analyse spoligotype data of the *M*. *tuberculosis* isolates [39–40]. For minimum spanning tree analysis the the Kruskal’s algorithm and force-directed graph layout for visualization according to the SIT using one single locus variation from the Node, SLV(1) was used to generate groupings into clonal clusters (CC) and Singletons (non-grouped) (Table 1).

^2^ Statistical models: generalized linear model, *Tuberculosis with a Euro-American lineage strain ~ HIV infection + MDRTB + gender + age group*, and *Tuberculosis with strains from MIRU-VNTR-plus spoligotype derived clonal complexes ~ HIV infection + MDRTB + gender + age group*

Note: Significance codes: 0 ‘***’ 0.001 ‘**’ 0.01 ‘*’ 0.05 ‘.’ 0.1 ‘ ‘ 1

MDRTB = multidrug resistant tuberculosis

The degree of resolution of the genotypic patterns observed was further increased in subsequent analysis using our earlier classification of locally adapted sympatric strains classified according to SIT. From analysis of the S1 and S2 Tables the association of CC4 to HIV was attributed to SIT244 infected cases. As HIV related genotypes have been previously associated to allopatric strains [37], two subsequent analyses were considered in our final model, one in which SIT244 was included in the sympatric group of strains and the other where it was grouped with the allopatric. In the former case, a positive association between HIV infection and sympatric strains was observed. However, if SIT244 was excluded from the sympatric and included in the allopatric group of SITs neither a positive nor negative relationship was observed with HIV infection. However, a significant level of association of MDRTB with sympatric strains was observed in the analysis of the entire dataset (n = 547) (S5 Table) and, in order to avoid artifacts due to rare spoligotype patterns, in analysis restricted to the “major subgroup” (n = 339) (Table 3). The association was significant, whether SIT244 was included in the sympatric (p < 0.001) or the allopatric (p < 0.001) group and was not affected when HIV infected cases were excluded (p < 0.001). Thus, the association of sympatric strains to MDRTB was not dependent on HIV status.

**Table 3:** Logistic regression analysis^1^ for the “major subgroup” of patients (n = 339) relative to tuberculosis caused by sympatric *M*. *tuberculosis* strains.

	Sympatric strains with SITs 20, 64, 389, 244, 1106 (n = 148).						
	Estimate	Standard Error	Z value	p-value	Odds Ratio	CI Lower Limit	CI Upper Limit
Constant	-14.566	882.743	-0.017	0.987			
MDRTB	2.155	0.558	3.857	<0.001 ***	8.629	3.191	30.183
HIV	0.659	0.278	2.365	0.018 *	1.933	1.121	3.355
	Sympatric strains with SITs 20, 64, 389, 1106 (n = 118).						
	Estimate	Standard Error	Z value	p-value	Odds Ratio	CI Lower Limit	CI Upper Limit
Constant	-14.566	882.743	-0.017	0.987			
MDRTB	2.250	0.488	4.607	<0.001 ***	9.487	3.859	26.991

^1^ Statistical model: generalized linear model, *Tuberculosis with a sympatric strain ~ HIV infection + MDRTB + gender + age group*

Note: The interactions between factors were tested but because they were not significant they were not included in the final model. Age and sex were not significant.

Note: Significance codes: 0 ‘***’ 0.001 ‘**’ 0.01 ‘*’ 0.05 ‘.’ 0.1 ‘ ’ 1

MDRTB = multidrug resistant tuberculosis

**Fig 2:**
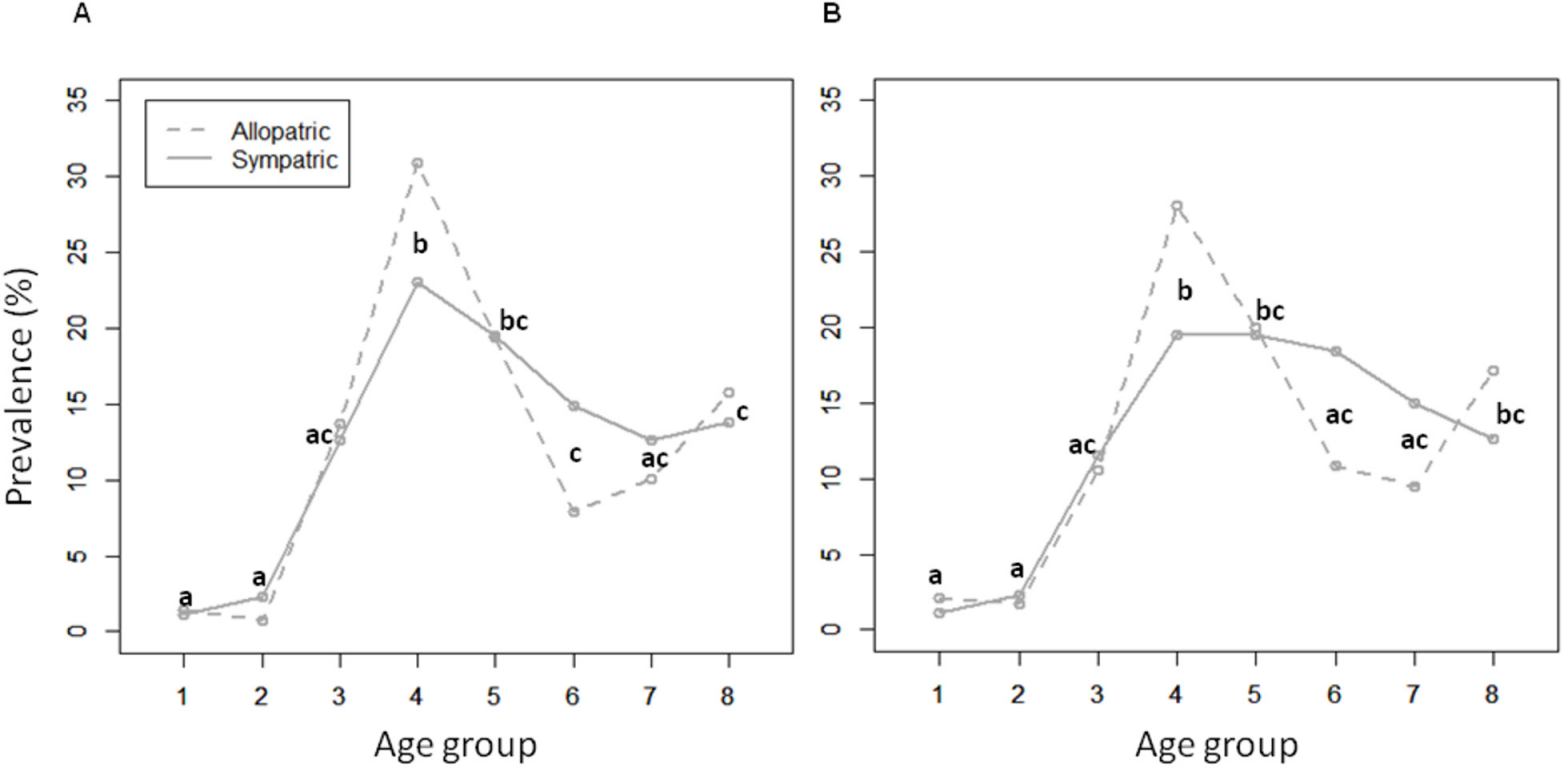
Distribution of the frequency of TB caused by sympatric and allopatric *M*. *tuberculosis* strains, as a function of the age group in HIV negative individuals, 68.0% (372/547). Groups 1 to 8 represent age categories in years of 0 to 4, 5 to 14, 15 to 24, 25 to 34, 35 to 44, 45 to 54, 55 to 64, over 65, respectively. Significant differences across the age groups (Tukey´s HSD, p<0.01) are indicated by lower case letters, identical when the same relationship is obtained; (A) TB by sympatric 26.3% (98/372) *versus* allopatric 73.7% (274/372) strains, marking the deviation from the usual trimodal age distribution curves; (B) TB by sympatric 23.4% (87/372) *versus* allopatric 76.6% (285/372) strains when excluding SIT244 strains as sympatric. Significant differences across Age group factor (Tukey´s HSD, p<0.01) are indicated by different letters being the same for each PTSIT (allopatric and sympatric).

For allopatric tuberculosis, prevalence distribution according to age showed three peaks, corresponding to the very young children, young adults and the elderly, generally referred to as a trimodal curve (Fig 2). Prevalence differences in age groups were not so pronounced for TB caused by sympatric strains due to a drop in prevalence in the very young and elderly, and alterations of the central peak. This was translated into a tendency towards a typical (standard) distribution curve. This tendency was further accentuated when SIT244 was included in the allopatric group of strains.

## Discussion

Portugal has remained endemic for TB, with higher incidence in large urban areas. Corroborating a slow decline of TB, the average age of incidence has increased [43]. During the time frame of this investigation, the estimated incidence of 28.2 cases per 100,000 inhabitants remained relatively high compared to the average of 17 cases per 100,000 inhabitants reported for the other European countries [43–44]. The highest reported incidence was of 45.8 per 100,000 in the Porto district followed by Lisbon with 35.5 per 100,000. In this setting, an important epidemiological concern was the 15% rate of TB/HIV co-infection, representing by far Europe’s highest, knowing that this level was certainly under reported [43,45]. HIV susceptibility has been related in Portugal and other southern European countries to a genetic background [46].

Lineage attribution based on whole genome LSP analysis considered six main *M*. *tuberculosis* phylogenetic lineages associated with different geographic regions [6–7]. This approach however appears less discriminative than the spoligotyping approach since the latter is able to resolve clinical isolates within the branch of the modern strains that are not solved by LSP. Although a better understanding of the *M*. *tuberculosis* population structure may be obtained with the concomitant use of several genotyping methods, spoligotyping alone is considered a tool for classification of strains within the Euro-American lineage [47]. Herein, spoligotyping allowed a smaller grain of resolution for the designation of sympatric strains in association analyses where other authors used the Euro-American lineage as a whole [37].

Within the Euro-American Lineage, spoligotype defined LAM strains have accounted for the vast majority of the cases in settings related to the Portuguese and Spanish colonization history. This family is particularly prevalent in the Mediterranean region and Latin America. From our previous findings it appears that Portugal may have one of the highest global proportions of LAM endemic strains [23,25]. Spoligotyping data restricted to the metropolitan area of Lisbon revealed a 51% prevalence of the LAM family, and a high proportion of SIT20 (LAM1) and SIT42 (LAM9) sub-families [23]. Furthermore, a major deletion, RD^Rio^, characteristic of LAM1 strains, although also associated with other LAM sub-families, was shown to have particular expression in settings related to early Iberian overseas expansion [18,25,47–49]. Interestingly, these strains were associated with ongoing transmission and higher bacillary load [50–51].

Accordingly, with reference to previous studies [23], *M*. *tuberculosis* LAM patterns related to the Portuguese population were considered as belonging to the sympatric group of strains, whereas others without this specific distribution were designated as allopatric. Therefore, considering the population structure specificities in our sample, characterized by a high prevalence of the LAM family, it was not unexpected that we should identify both allopatric and sympatric strains within the coarser grain of resolution of the Euro-American lineage. Possibly due to this overlap, we did not observe a significant relationship between the Euro-American lineage and HIV infection. We further investigated the statistical relevance of the association of sympatric strains with regards to HIV infection, age, gender and MDR TB at a finer grain of analysis, using MIRU-VNTRplus spoligotype phylogenetic clusters. Association of HIV with the spoligotype clonal complex containing SIT244 was evidenced (p < 0.001). This T spoligotype family genotype, contrary to the LAM sympatric genotypes, was not detected in the independent convenient samples from other regions in Portugal. These facts contributed to rebuttal against the classification of this SIT244 amongst the sympatric strains. Moreover, in the SpolDB4, database SIT244 was important in only two settings, Portugal and Bangladesh [15,52]. Portuguese traders and missionaries were the first Europeans to settle in Bengal (what is now known as Bangladesh) as far back as the 15th century. In the sixteenth century Chittagong (Porto Grande) was a thriving Portuguese and Eurasian community of over 5000 people. The Portuguese presence remained in the region until the mid-seventeenth century when they were forced to leave by local opposition. Therefore, the nature of ties between the two populations, limited to a particular socioeconomical sector of the population during a specific time frame would unlikely have resulted in continuous sharing of genetic features. The SIT244 may have resulted from an imported allopatric strain that ravished amongst HIV infected patients in the Lisbon area.

Consequently, eliminating SIT244 from the sympatric group of strains our results corroborated previous findings in that TB with sympatric strains was not associated with HIV infection [37]. Although a high proportion of SIT244 strains was found in HIV-infected patients, no association of the allopatric strains including this SIT with HIV status was demonstrated. The finding, according to Fenner and collaborators [37], that HIV associates preferentially with allopatric strains was not confirmed in our setting when considering the non Euro-American lineage strains as allopatric versus the Euro-American lineage as sympatric. The association of HIV to MIRU-VNTR spoligotype based clonal complex CC4 was evidenced at a greater degree of genotypic resolution than lineage. Our findings do not invalidate the conclusion of Fenner and collaborators [37], but highlighted the necessity of zooming in on the genotypic resolution for detecting relevant associations.

A significant level of association of MDRTB with sympatric strains, was found. Sympatric strains are prone to outperform allopatric pathogens namely with increased and frequent ongoing transmission compared to allopatric host–pathogen combinations [7,37]. The major spoligotype signature SIT20 in our study belongs to the LAM1 sub-family. Also, in our previous studies, the RD^Rio^ deletion was detected in up to 60% of the LAM isolates in Portugal and was associated with MDR and XDR transmission clusters [25,53]. So, although active transmission of isolates from this study was not evaluated, the active transmission of major LAM spoligotypes relevant to this study was available from other reports [22,25,48,53].

Additionally, the statically significant association with male gender, observed for two clonal complexes, has been previously described in this and other settings. This tendency has been described as characteristic of disease endemicity [43] although the reasons remain unclear [54].

In order to further qualify the host pathogen relationship, distribution curves of prevalence in relation to patients’ age were analyzed with regards to the sympatric and allopatric host pathogen relationship in HIV negative patients. The typical trimodal curve has previously proven useful as a marker of the epidemic evolution of the disease, showing a gradual peak shift to older persons [4], also observed in Portugal, in the decade of 1997 to 2006 [43]. Age related peaks in the lower and upper extremes of disease prevalence distribution curves have been typically attributed to impaired immunity, and the central peak to socioeconomic factors. At a higher age, the increase of cases is frequently attributed to reactivation. Although new cases *versus* reactivation was not assessed in this age group, this idea has come to be questioned in other settings since the generalized application of molecular epidemiological tools, revealing a high proportion of clustered isolates suggestive of recent transmission [55–58].

The trimodal distribution mentioned above was accentuated in the case of allopatric genotypes (Fig 2A) and maintained when SIT244 was included in this group of strains (Fig 2B). However, for TB caused by sympatric strains, a tendency towards a typical (standard) distribution curve was observed. It would appear that age is less relevant for susceptibility in the case of these highly transmissible sympatric strains.

On the other hand, the human genetics approach to mycobacterial infections has brought new insight to the genetic predisposition to disease with practical implications towards the development of a personalized medicine [59–60]. Genetic association studies represent a powerful tool in the identification of host genetic variants implicated in infection and disease and have given proof of evidence of the association between clinical disease and genes of both the innate or adaptive anti-mycobacterial immunity [59–68]. This approach is being increasingly discussed in terms of the host-pathogen relationship, where pathogen genotype is also considered [29–33].

Clinical onset at an early age has been attributed to rare mutations responsible Monogenic Mendelian traits and late onset to common polymorphisms having a milder effect on the risk of clinical disease. These however, have been considered as the two ends of a continuous spectrum of genetic susceptibility. Ensuring this continuum we would find relatively rare variants having a major gene effect [62,64]. Relative to the allopatric strains, we may assume that there has been less exposure, translated by an accentuation of the relevance of the age at risk as reflected in the trimodal distribution for TB prevalence. On the contrary, for the sympatric strains, the greater stability of host-pathogen relationship, characteristic of populations having survived historical exposure, the attenuation of the central peak and loss of the peaks at the extremes of the age distribution could be suggestive of genetic determinisms due to more common polymorphisms. Moreover, a major gene effect governing the host-pathogen sympatric relationship as suggested by these results from our particular study setting would be concordant with aspects governing the geographic specificity of Europe’s human genetic variation [69] and appears particularly relevant for future study design.

An important strength of our study was the availability of large dataset resulting from the length of the sampling period (7 years) and the use of a consecutive mode of sampling of one isolate per TB patient from the major hospital deserving one of the most densely populated urban areas of Lisbon. Information on country of birth (or ancestry) was not available from our data set. To counter this fault, we relied on nationwide surveys reported by the Portuguese National School of Public Health referent to the same time frame as this study indicating that the incidence and endemic levels of TB in Portugal are mainly due to TB in nationals, contrary to what has been observed in some of the Northern European countries [43,45,70]. Generally speaking, a patient’s country of birth has been used as a surrogate of the ancestry of the study population in *M*. *tuberculosis* phylogeography [8,34,37]. However, this does not apply to our setting as the wave of immigration in Portugal dates to decolonization in the 1970’s so these populations now constitute Portuguese born second and third generations.

In TB, molecular epidemiological evidence suggestive of host-pathogen co-evolution has allowed pathogen genotypes to be grouped into distinct lineages in a way that correlates with ethnicity and geography. A central aspect of this analysis is the introduction of the idea that differences in the immunological response of the host can vary in accordance to the infectious strain. This premise has important implications, namely suggesting there could be a need for different vaccines in different parts of the world (precision medicine). Less virulent strains can be associated with immune deficiencies. As a working hypothesis, allopatric strains could be successful against a host background with less history of exposure and higher vulnerability. Moreover, populations less fitted to the epidemic strains of the industrial revolution would have given way to the modern Europeans adapted to their sympatric strains. In terms of public health, the question of the duration of sympatric strain transmission clusters which, as we have seen, may be associated with severe MDRTB cases, remains unanswered. The reduction in number of TB cases accompanied by the steady rise of the age of the at-risk group, as observed on national scales, reflects the decline of an earlier epidemic. Would this be consistent with the view of coevolution between *M*. *tuberculosis* and its human host? How could the consequences that may be expected in terms of the genetic predisposition to the disease be translated into testable models using complementary approaches to provide the needed insight to deal with modern TB epidemics?

## Conclusions

Increased recognition of the phylogeographic distribution of *M*. *tuberculosis* has brought much attention to pathogen genotype and how it may articulate with the host genetic background in the host pathogen-relationship. *M*. *tuberculosis* strains can be classified within sympatric or allopatric host-pathogen relationships at the discretion of genotypic resolution. Here, we argue that considering the *M*. *tuberculosis* Euro-American lineage as a whole, ignoring lineage subdivisions into genotypic families as published by Fenner and collaborators [37], may compromise future assumptions in this type of approach. Associations with clinical and demographic factors were evidenced using more discriminative strain genotyping, not being discernible at the lineage level. The impact on modeling divergence was also reflected by the study of the distribution of genotype prevalence among different age groups. This was discussed in terms of host genetic predisposition to disease, which may have important consequences in the design of genetic association studies. The choice of determinants in the definition of sympatric or allopatric host-pathogen relationships may thus affect working models of infection and disease in TB and, likewise, the interpretation of epidemiology and clinical outcome, with important implications in dealing with the modern TB epidemics.

## Supporting Information

S1 Table Data set of patients and *M*. *tuberculosis isolates* from this study (n = 547). (XLSX)

S2 Table Molecular analysis of the *M*. *tuberculosis* isolates from the patients in this study (n = 547). (PDF)

S3 Table Description of the major spoligotypes (representing two or more isolates) of the convenient sample from the district of Lisbon. (DOC)

S4 Table Description of the major spoligotypes (representing two or more isolates) of the convenient sample from the district of Porto. (DOC)

S5 Table Logistic regression analysis of TB cases caused by sympatric *M*. *tuberculosis* strains. (DOC)
