# Supplementary material for: Determinants of the Sympatric Host-Pathogen Relationship in Tuberculosis
Source: PLoS One. 2015 Nov 3;10(11):e0140625. doi: 10.1371/journal.pone.0140625 (PMC4631367; doi:10.1371/journal.pone.0140625)
Supplement: S2 Table — (PDF) [file pone.0140625.s002.pdf]

Table S2. Molecular analysis of the *M. tuberculosis* isolates from the patients in this study (n = 547)

Spoligotype analysis			MIRU-VNTRplus clonal complexes[3]								Euro-American lineage[4]	Number of cases
SIT[1]	Spoligotype	Label according to SPOTCLUST[2]	CC1	CC2	CC3	CC4	CC5	CC6-CC19	Singletons	Clustered		
1		Beijing	0	0	0	0	0	0	1	0	0	15
NC		CAS	0	0	0	0	0	0	1	0	0	1
26		CAS	0	0	0	0	0	0	1	0	0	2
NC		EAI1 99%	0	0	0	0	0	0	1	0	0	1
NC		EAI1 99%	0	0	0	0	0	0	1	0	0	1
NC		EAI2 68% EAI5 31%	0	0	0	0	0	0	1	0	0	1
129		EAI5	0	0	0	0	0	0	1	0	0	1
1251		EAI5	0	0	0	0	0	0	1	0	0	1
NC		Family33	0	0	0	0	0	0	1	0	0	1
523		Family33	0	0	0	0	0	0	1	0	0	3
NC		Family34 96%	0	0	0	0	0	1	0	1	0	1
NC		Family34 99%	0	0	0	0	0	0	1	0	0	1
402		Family34	0	0	0	0	0	1	0	1	0	1
1757		Family34 89% EAI1 11%	0	0	0	0	0	1	0	1	0	3
NC		Family36	0	0	0	0	0	0	1	0	0	1
4		Family36	0	0	0	0	0	1	0	1	0	4
635		Family36	0	0	0	0	0	1	0	1	0	1
451		H37Rv 74% T1 26%	0	0	0	0	0	0	1	0	1	1
45		H1	0	0	0	0	1	0	0	1	1	1
47		H1	0	0	0	0	1	0	0	1	1	21
62		H1	0	0	0	0	1	0	0	1	1	1
151		H1	0	0	0	0	1	0	0	1	1	2
NC		H3 77% T1 23%	0	0	0	0	0	0	1	0	1	1
49		H3 72% T1 28%	0	1	0	0	0	0	0	1	1	3
50		H3 77% T1 23%	0	1	0	0	0	0	0	1	1	13
75		H3 77% T1 23%	0	1	0	0	0	0	0	1	1	1
262		H3 77% T1 23%	0	0	0	0	0	0	1	0	1	1
293		H3 77% T1 23%	0	1	0	0	0	0	0	1	1	1
448		H3 72% T1 28%	0	1	0	0	0	0	0	1	1	1
746		H3 77% T1 23%	0	1	0	0	0	0	0	1	1	1
NC		LAM1 66% LAM9 34%	1	0	0	0	0	0	0	1	1	1
NC		LAM1 66% LAM9 34%	0	0	0	0	0	0	1	0	1	1
NC		LAM1 66% LAM9 34%	1	0	0	0	0	0	0	1	1	1
NC		LAM1 66% LAM9 34%	0	0	0	0	0	0	1	0	1	1
NC		LAM1 66% LAM9 34%	0	0	0	0	0	0	1	0	1	1
NC		LAM1 66% LAM9 34%	1	0	0	0	0	0	0	1	1	1
NC		LAM1 66% LAM9 34%	1	0	0	0	0	0	0	1	1	1
NC		LAM1 66% LAM9 34%	0	0	0	0	0	0	1	0	1	2
NC		LAM1 66% LAM9 34%	0	0	0	0	0	0	1	0	1	1
NC		LAM1 69% LAM9 31%	0	0	0	0	0	0	1	0	1	1
NC		LAM1 69% LAM9 31%	0	0	0	0	0	0	1	0	1	1
20		LAM1 66% LAM9 34%	1	0	0	0	0	0	0	1	1	82
389		LAM1 66% LAM9 34%	1	0	0	0	0	0	0	1	1	13
1249		LAM1 69% LAM9 31%	0	0	0	0	0	0	1	0	1	1
1321		LAM1 66% LAM9 34%	1	0	0	0	0	0	0	1	1	3
1752		LAM1 66% LAM9 34%	1	0	0	0	0	0	0	1	1	5
1755		LAM1 51% LAM9 49%	1	0	0	0	0	0	0	1	1	2
17		LAM2 82% LAM1 11%	1	0	0	0	0	0	0	1	1	10
545		LAM2 82% LAM1 11%	1	0	0	0	0	0	0	1	1	1
NC		LAM3 99%	0	0	0	0	0	0	1	0	1	1
NC		LAM3 99%	0	0	0	0	0	1	0	1	1	1
33		LAM3	0	0	0	0	0	1	0	1	1	6
111		LAM3	0	0	0	0	0	1	0	1	1	1
211		LAM3 99%	0	0	0	0	0	1	0	1	1	8
1759		LAM3	0	0	0	0	0	1	0	1	1	4
1760		LAM3 99%	0	0	0	0	0	0	1	0	1	1
29		LAM7	0	0	0	0	0	0	1	0	1	2
106		LAM7	0	0	0	0	0	1	0	1	1	1
NC		LAM8	0	0	0	0	0	0	1	0	1	1
NC		LAM8	0	0	0	0	0	0	1	0	1	1
NC		LAM8	0	0	0	0	0	1	0	1	1	1
NC		LAM8 91%	0	0	0	0	0	1	0	1	1	1
NC		LAM8 97%	0	0	0	0	0	0	1	0	1	1
NC		LAM8 99%	0	0	0	0	0	1	0	1	1	1
59		LAM8 94%	0	0	0	0	0	1	0	1	1	1
283		LAM8 87% H1 13%	0	0	0	0	0	0	1	0	1	1
397		LAM8	0	0	0	0	0	0	1	0	1	3
NC		LAM9	0	0	0	0	0	0	1	0	1	1
NC		LAM9	0	0	0	0	0	0	1	0	1	1
NC		LAM9	0	0	0	0	0	1	0	1	1	1
NC		LAM9	0	0	0	0	0	1	0	1	1	1
NC		LAM9	0	0	0	0	0	0	1	0	1	1
NC		LAM9	1	0	0	0	0	0	0	1	1	1
NC		LAM9	0	0	0	0	0	0	1	0	1	1
NC		LAM9	0	0	0	0	0	0	1	0	1	1
NC		LAM9 60% T2 40%	0	0	0	0	0	0	1	0	1	1
NC		LAM9 98%	0	0	0	0	0	0	1	0	1	1
42		LAM9	1	0	0	0	0	0	0	1	1	61
60		LAM9	1	0	0	0	0	0	0	1	1	8
64		LAM9	1	0	0	0	0	0	0	1	1	14
81		LAM9	1	0	0	0	0	0	0	1	1	5
93		LAM9	1	0	0	0	0	0	0	1	1	1
150		LAM9	1	0	0	0	0	0	0	1	1	20
161		LAM9	1	0	0	0	0	0	0	1	1	1
177		LAM9	1	0	0	0	0	0	0	1	1	1
396		LAM9	1	0	0	0	0	0	0	1	1	1
766		LAM9 97%	0	0	0	0	0	0	1	0	1	1
1064		LAM9	0	0	0	0	0	1	0	1	1	1
1106		LAM9 60% T2 40%	0	0	0	0	0	0	1	0	1	9
1750		LAM9	1	0	0	0	0	0	0	1	1	2
1758		LAM9	1	0	0	0	0	0	0	1	1	1
61		LAM10 98%	0	0	0	0	0	1	0	1	1	1
115		LAM10 98%	0	0	0	0	0	1	0	1	1	1
181		*M. africanum*	0	0	0	0	0	1	0	1	0	3
324		*M. africanum*	0	0	0	0	0	1	0	1	0	1
NC		S 98%	0	0	1	0	0	0	0	1	1	1
34		S 78% T1 22%	0	0	1	0	0	0	0	1	1	12
156		S 93%	0	0	1	0	0	0	0	1	1	1
784		S 81% T1 19%	0	0	1	0	0	0	0	1	1	1
NC		T1	0	0	0	0	0	0	1	0	1	1
NC		T1	0	0	0	0	0	0	1	0	1	1
NC		T1	0	0	0	0	0	0	1	0	1	1
NC		T1	0	0	0	0	0	0	1	0	1	1
NC		T1	0	0	0	0	0	0	1	0	1	1
NC		T1 93%	0	0	0	1	0	0	0	1	1	1
37		T1	0	1	0	0	0	0	0	1	1	1
44		T1	0	1	0	0	0	0	0	1	1	2
53		T1	0	1	0	0	0	0	0	1	1	39
58		T1	0	1	0	0	0	0	0	1	1	3
73		T1	0	1	0	0	0	0	0	1	1	6
118		T1	0	1	0	0	0	0	0	1	1	7
154		T1	0	1	0	0	0	0	0	1	1	2
157		T1	0	0	0	0	0	0	1	0	1	4
462		T1	0	1	0	0	0	0	0	1	1	1
521		T1 93%	0	0	0	1	0	0	0	1	1	1
732		T1	0	1	0	0	0	0	0	1	1	2
787		T1	0	0	0	0	0	0	1	0	1	2
834		T1	0	1	0	0	0	0	0	1	1	2
1196		T1 99%	0	1	0	0	0	0	0	1	1	1
NC		T2 82% T1 18%	0	0	0	1	0	0	0	1	1	1
NC		T2 82% T1 18%	0	0	0	0	0	0	1	0	1	1
NC		T2 82% T1 18%	0	0	0	0	0	0	1	0	1	1
NC		T2 82% T1 18%	0	0	0	0	0	0	1	0	1	2
NC		T2 98%	0	0	0	0	0	1	0	1	1	2
51		T2 98%	0	0	0	0	0	1	0	1	1	4
237		T2	0	0	0	0	0	0	1	0	1	1
244		T2 82% T1 18%	0	0	0	1	0	0	0	1	1	30
NC		T3	0	0	0	0	0	0	1	0	1	1
144		T3 99%	0	0	0	0	0	0	1	0	1	1
92		X3	0	0	0	0	0	1	0	1	1	11
344		X3	0	0	0	0	0	1	0	1	1	3
1756		X3	0	0	0	0	0	0	1	0	1	1

[1] Shared International Type (SIT), International spoligotype database SpolDB4 (http://www.pasteur-guadeloupe.fr:8081/SITVITdemo/) [15].

[2] Label representing spoligotype families as assigned in the SPOTCLUST program (http://cgi2.cs.rpi.edu/~bennek/SPOTCLUST.html) [38].

[3] The MIRU-VNTRplus web application (http://www.miru-vntrplus.org) was used to analyze spoligotype data of the *M. tuberculosis* isolates [39-40]. The minimum spanning tree analysis according to the SIT using one single locus variation from the Node, SLV(1) was used to generate groupings into clonal clusters (CC).

[4] *M. tuberculosis* lineage designation, Euro-American, was according to Gagneux and collaborators [7].
