# Supplementary material for: Determinants of the Sympatric Host-Pathogen Relationship in Tuberculosis
Source: PLoS One. 2015 Nov 3;10(11):e0140625. doi: 10.1371/journal.pone.0140625 (PMC4631367; doi:10.1371/journal.pone.0140625)
Supplement: S3 Table — (DOC) [file pone.0140625.s003.doc]

**Table S3. Description of the major spoligotypes (representing two or more isolates) of the convenient sample from the district of Lisbon^1^**

| **SIT**^2^ | **Spoligotype** | **Label**^3^ **according to** | | **Nº of Isolates**^4^ | **Prevalence (%)** |
| --- | --- | --- | --- | --- | --- |
|  |  | **SPOTCLUST** | **SpolDB4** |  |  |
| 20 | ⬛⬛⬜⬛⬛⬛⬛⬛⬛⬛⬛⬛⬛⬛⬛⬛⬛⬛⬛⬛⬜⬜⬜⬜⬛⬛⬛⬛⬛⬛⬛⬛⬜⬜⬜⬜⬛⬛⬛⬛⬛⬛⬛ | LAM1 66% LAM9 34% | LAM1 | 29 | 21.5 |
| 42 | ⬛⬛⬛⬛⬛⬛⬛⬛⬛⬛⬛⬛⬛⬛⬛⬛⬛⬛⬛⬛⬜⬜⬜⬜⬛⬛⬛⬛⬛⬛⬛⬛⬜⬜⬜⬜⬛⬛⬛⬛⬛⬛⬛ | LAM9 | LAM9 | 15 | 11.1 |
| 1106 | ⬛⬛⬛⬛⬛⬛⬛⬛⬛⬛⬛⬛⬛⬛⬛⬛⬛⬛⬛⬛⬜⬜⬜⬜⬛⬛⬛⬛⬛⬛⬛⬛⬜⬜⬜⬜⬛⬜⬜⬜⬜⬜⬜ | LAM9 60% T2 40% | LAM9 | 8 | 5.9 |
| 17 | ⬛⬛⬜⬛⬛⬛⬛⬛⬛⬛⬛⬛⬜⬛⬛⬛⬛⬛⬛⬛⬜⬜⬜⬜⬛⬛⬛⬛⬛⬛⬛⬛⬜⬜⬜⬜⬛⬛⬛⬛⬛⬛⬛ | LAM2 82% LAM1 11% | LAM2 | 5 | 3.7 |
| 73 | ⬛⬛⬛⬛⬛⬛⬛⬛⬛⬛⬛⬛⬜⬛⬛⬛⬛⬛⬛⬛⬛⬛⬛⬛⬛⬛⬛⬛⬛⬛⬛⬛⬜⬜⬜⬜⬛⬛⬛⬜⬛⬛⬛ | T1 | T2+T3 | 5 | 3.7 |
| 1 | ⬜⬜⬜⬜⬜⬜⬜⬜⬜⬜⬜⬜⬜⬜⬜⬜⬜⬜⬜⬜⬜⬜⬜⬜⬜⬜⬜⬜⬜⬜⬜⬜⬜⬜⬛⬛⬛⬛⬛⬛⬛⬛⬛ | Beijing | Beijing | 5 | 3.7 |
| 47 | ⬛⬛⬛⬛⬛⬛⬛⬛⬛⬛⬛⬛⬛⬛⬛⬛⬛⬛⬛⬛⬛⬛⬛⬛⬛⬜⬜⬜⬜⬜⬜⬛⬜⬜⬜⬜⬛⬛⬛⬛⬛⬛⬛ | Haarlem1 | H1 | 4 | 3.0 |
| 49 | ⬛⬛⬛⬛⬛⬛⬛⬛⬛⬛⬛⬛⬛⬛⬛⬛⬛⬛⬛⬛⬛⬛⬛⬛⬛⬛⬛⬛⬛⬛⬜⬛⬜⬜⬜⬜⬛⬛⬛⬛⬛⬛⬛ | Haarlem3 77% T1 23% | H3 | 4 | 3.0 |
| 891 | ⬛⬛⬛⬛⬛⬛⬛⬛⬛⬛⬛⬛⬛⬛⬛⬛⬛⬛⬛⬛⬜⬜⬜⬜⬛⬛⬛⬛⬛⬜⬛⬛⬜⬜⬜⬜⬛⬛⬛⬛⬛⬛⬛ | LAM9 | LAM9 | 3 | 2.2 |
| 578 | ⬛⬛⬜⬜⬛⬛⬛⬛⬛⬛⬛⬛⬛⬛⬛⬛⬛⬛⬛⬛⬜⬜⬜⬜⬛⬛⬛⬛⬛⬛⬛⬛⬜⬜⬜⬜⬛⬛⬛⬛⬛⬛⬛ | LAM1 66% LAM9 34% | LAM9 | 2 | 1.5 |
| 64 | ⬛⬛⬛⬛⬛⬛⬛⬛⬛⬛⬛⬛⬛⬛⬛⬛⬛⬛⬛⬛⬜⬜⬜⬜⬛⬛⬛⬛⬜⬛⬛⬛⬜⬜⬜⬜⬛⬛⬛⬛⬛⬛⬛ | LAM9 | LAM6 | 2 | 1.5 |
| 60 | ⬛⬛⬛⬛⬛⬛⬛⬛⬛⬛⬛⬛⬛⬛⬛⬛⬛⬛⬛⬛⬜⬜⬜⬜⬛⬛⬛⬛⬛⬛⬛⬛⬜⬜⬜⬜⬛⬛⬛⬜⬛⬛⬛ | LAM9 | LAM4 | 2 | 1.5 |
| 291 | ⬛⬛⬛⬛⬛⬛⬛⬛⬛⬛⬛⬛⬛⬛⬛⬛⬛⬛⬛⬛⬜⬛⬛⬛⬛⬛⬛⬛⬛⬛⬛⬛⬜⬜⬜⬜⬛⬛⬛⬛⬛⬛⬛ | T1 | T1 | 2 | 1.5 |
| 53 | ⬛⬛⬛⬛⬛⬛⬛⬛⬛⬛⬛⬛⬛⬛⬛⬛⬛⬛⬛⬛⬛⬛⬛⬛⬛⬛⬛⬛⬛⬛⬛⬛⬜⬜⬜⬜⬛⬛⬛⬛⬛⬛⬛ | T1 | T1 | 2 | 1.5 |
| 137 | ⬛⬛⬛⬛⬛⬛⬛⬛⬛⬛⬛⬛⬛⬛⬛⬛⬛⬜⬛⬛⬛⬛⬛⬛⬛⬛⬛⬛⬛⬛⬛⬛⬜⬜⬜⬜⬛⬛⬜⬜⬜⬜⬛ | X2 | X2 | 2 | 1.5 |
| 225 | ⬛⬛⬛⬛⬛⬛⬛⬛⬛⬛⬛⬛⬛⬛⬛⬛⬛⬜⬛⬛⬛⬛⬛⬛⬛⬛⬛⬛⬛⬛⬛⬛⬜⬜⬜⬜⬛⬛⬜⬜⬜⬛⬛ | X1 57% T1 40% | X1 | 2 | 1.5 |
| NA | ⬛⬛⬛⬜⬜⬜⬜⬜⬜⬜⬜⬜⬛⬛⬛⬛⬜⬜⬛⬛⬛⬛⬛⬛⬛⬛⬛⬛⬛⬛⬛⬛⬜⬜⬜⬜⬛⬛⬛⬜⬛⬛⬛ | X3 | NA | 2 | 1.5 |
| NA | ⬛⬛⬛⬜⬜⬜⬜⬜⬜⬜⬜⬜⬛⬛⬛⬛⬜⬜⬛⬛⬛⬛⬛⬛⬛⬛⬛⬛⬛⬛⬛⬛⬜⬜⬜⬜⬛⬛⬛⬛⬛⬛⬛ | X3 | NA | 2 | 1.5 |

^1^ Adapted from [69-70]

^2^ Shared International Type (SIT), International spoligotype database SpolDB4 (<http://www.pasteur-guadeloupe.fr:8081/SITVITdemo/>) [15].

^3^ Label representing spoligotype families as assigned in the International spoligotype database SpolDB4 and by the SPOTCLUST program (<http://cgi2.cs.rpi.edu/~bennek/SPOTCLUST.html>) [38].

^4^ Number of isolates with a particular spoligotype relative to the total number of isolates from the district of Lisbon, in percentile.
