# Supplementary material for: Determinants of the Sympatric Host-Pathogen Relationship in Tuberculosis
Source: PLoS One. 2015 Nov 3;10(11):e0140625. doi: 10.1371/journal.pone.0140625 (PMC4631367; doi:10.1371/journal.pone.0140625)
Supplement: S4 Table — (DOC) [file pone.0140625.s004.doc]

**Table S4. Description of the major spoligotypes (representing two or more isolates) of the convenient sample from the district of Porto^1^**

| **SIT**^2^ | **Spoligotype** | **Label**^3^ **according to** | | **Nº of Isolates**^4^ | **Prevalence**  **(%)** |
| --- | --- | --- | --- | --- | --- |
|  |  | **SPOTCLUST** | **SpolDB4** |  |  |
| 20 | ⬛⬛⬜⬛⬛⬛⬛⬛⬛⬛⬛⬛⬛⬛⬛⬛⬛⬛⬛⬛⬜⬜⬜⬜⬛⬛⬛⬛⬛⬛⬛⬛⬜⬜⬜⬜⬛⬛⬛⬛⬛⬛⬛ | LAM1 66% LAM9 34% | LAM1 | 11 | 16.9 |
| 42 | ⬛⬛⬛⬛⬛⬛⬛⬛⬛⬛⬛⬛⬛⬛⬛⬛⬛⬛⬛⬛⬜⬜⬜⬜⬛⬛⬛⬛⬛⬛⬛⬛⬜⬜⬜⬜⬛⬛⬛⬛⬛⬛⬛ | LAM9 | LAM9 | 4 | 6.2 |
| NA | ⬜⬜⬛⬛⬛⬛⬛⬛⬜⬜⬜⬛⬜⬛⬛⬛⬛⬛⬛⬛⬜⬜⬜⬜⬛⬛⬛⬛⬛⬛⬛⬛⬜⬜⬜⬜⬛⬛⬛⬛⬛⬛⬛ | LAM3 99% | NA | 4 | 6.2 |
| 33 | ⬛⬛⬛⬛⬛⬛⬛⬛⬜⬜⬜⬛⬛⬛⬛⬛⬛⬛⬛⬛⬜⬜⬜⬜⬛⬛⬛⬛⬛⬛⬛⬛⬜⬜⬜⬜⬛⬛⬛⬛⬛⬛⬛ | LAM3 | LAM3 | 3 | 4.6 |
| 1750 | ⬛⬛⬛⬛⬛⬛⬛⬛⬛⬛⬛⬛⬛⬛⬜⬛⬛⬛⬛⬛⬜⬜⬜⬜⬛⬛⬛⬛⬛⬛⬛⬛⬜⬜⬜⬜⬛⬛⬛⬜⬛⬛⬛ | LAM9 | LAM4 | 3 | 4.6 |
| 389 | ⬛⬛⬜⬛⬛⬛⬛⬛⬛⬛⬛⬛⬛⬛⬜⬛⬛⬛⬛⬛⬜⬜⬜⬜⬛⬛⬛⬛⬛⬛⬛⬛⬜⬜⬜⬜⬛⬛⬛⬛⬛⬛⬛ | LAM1 66% LAM9 34% | LAM1 | 3 | 4.6 |
| 58 | ⬛⬛⬛⬛⬛⬛⬛⬛⬛⬛⬛⬛⬛⬛⬛⬛⬛⬛⬛⬜⬛⬛⬜⬛⬛⬛⬛⬛⬛⬛⬛⬛⬜⬜⬜⬜⬛⬛⬛⬛⬛⬛⬛ | T1 | T5_MAD2 | 3 | 4.6 |
| 53 | ⬛⬛⬛⬛⬛⬛⬛⬛⬛⬛⬛⬛⬛⬛⬛⬛⬛⬛⬛⬛⬛⬛⬛⬛⬛⬛⬛⬛⬛⬛⬛⬛⬜⬜⬜⬜⬛⬛⬛⬛⬛⬛⬛ | T1 | T1 | 3 | 4.6 |
| 1759 | ⬛⬛⬛⬛⬛⬛⬜⬛⬜⬜⬜⬛⬛⬛⬛⬛⬛⬛⬛⬛⬜⬜⬜⬜⬛⬛⬜⬛⬛⬛⬛⬛⬜⬜⬜⬜⬛⬛⬛⬛⬛⬛⬛ | LAM3 | LAM8 | 2 | 3.1 |
| 822 | ⬛⬛⬛⬛⬜⬛⬛⬛⬛⬛⬛⬛⬛⬛⬛⬛⬛⬛⬛⬛⬜⬜⬜⬜⬛⬛⬛⬛⬛⬛⬛⬛⬜⬜⬜⬜⬛⬛⬛⬛⬛⬛⬛ | LAM9 | LAM9 | 2 | 3.1 |
| NA | ⬛⬛⬛⬛⬛⬛⬛⬜⬜⬜⬛⬛⬛⬛⬛⬛⬛⬛⬜⬛⬜⬜⬜⬜⬛⬛⬛⬛⬜⬛⬛⬛⬜⬜⬜⬜⬛⬛⬛⬛⬛⬛⬛ | LAM9 | NA | 2 | 3.1 |
| 73 | ⬛⬛⬛⬛⬛⬛⬛⬛⬛⬛⬛⬛⬜⬛⬛⬛⬛⬛⬛⬛⬛⬛⬛⬛⬛⬛⬛⬛⬛⬛⬛⬛⬜⬜⬜⬜⬛⬛⬛⬜⬛⬛⬛ | T1 | T2+T3 | 2 | 3.1 |
| 49 | ⬛⬛⬛⬛⬛⬛⬛⬛⬛⬛⬛⬛⬛⬛⬛⬛⬛⬛⬛⬛⬛⬛⬛⬛⬛⬛⬛⬛⬛⬛⬜⬛⬜⬜⬜⬜⬛⬛⬛⬛⬛⬛⬛ | Haarlem3 77% T1 23% | H3 | 2 | 3.1 |
| 47 | ⬛⬛⬛⬛⬛⬛⬛⬛⬛⬛⬛⬛⬛⬛⬛⬛⬛⬛⬛⬛⬛⬛⬛⬛⬛⬜⬜⬜⬜⬜⬜⬛⬜⬜⬜⬜⬛⬛⬛⬛⬛⬛⬛ | Haarlem1 | H1 | 2 | 3.1 |

^1^  Adapted from [69-70]

^2^ Shared International Type (SIT), International spoligotype database SpolDB4 (<http://www.pasteur-guadeloupe.fr:8081/SITVITdemo/>) [15].

^3^ Label representing spoligotype families as assigned in the International spoligotype database SpolDB4 and by the SPOTCLUST program (<http://cgi2.cs.rpi.edu/~bennek/SPOTCLUST.html>) [38].

^4^ Number of isolates with a particular spoligotype relative to the total number of isolates from the district of Porto, in percentile.
