# Supplementary material for: Determinants of the Sympatric Host-Pathogen Relationship in Tuberculosis
Source: PLoS One. 2015 Nov 3;10(11):e0140625. doi: 10.1371/journal.pone.0140625 (PMC4631367; doi:10.1371/journal.pone.0140625)
Supplement: S5 Table — (DOC) [file pone.0140625.s005.doc]

| **Table S5. Logistic regression analysis of tuberculosis patients (n = 547) ) relative to tuberculosis caused by sympatric *M. tuberculosis* strains** | | | | | | | |
| --- | --- | --- | --- | --- | --- | --- | --- |
|  | Sympatric strains with SITs 20, 64, 389, 244, 1106 (n = 148). | | | | | | |
|  | Estimate | Standard Error | Z value | p-value | Odds Ratio | CI  Lower Limit | CI  Upper Limit |
|  | | | | | | | |
| Constant | -1.186 | 0.134 | 8.872 | <0.001 *** |  |  |  |
| MDRTB | 1.821 | 0.393 | 4.632 | <0.001 *** | 6.175 | 2.937 | 13.931 |
| HIV | 0.584 | 0.216 | 2.706 | <0.01 ** | 1.792 | 1.173 | 2.734 |
|  |  |  |  |  |  |  |  |
|  | Sympatric strains with SITs 20, 64, 389, 1106 (n = 118). | | | | | | |
|  | Estimate | Standard Error | Z value | p-value | Odds Ratio | CI  Lower Limit | CI  Upper Limit |
| Constant | -1.668 | 0.237 | -7.033 | <0.001 *** |  |  |  |
| MDRTB | 1.989 | 0.378 | 5.268 | <0.001 *** | 1.988 | 1.266 | 2.759 |
| ^1^ Statistical model: generalized linear model, *Tuberculosis with a sympatric strain ~ HIV infection + MDRTB + gender + age group*  Note: The interactions between factors were tested but because they were not significant they were not included in the final model. Age and sex were not significant.  Note: Significance codes: 0 ‘***’ 0.001 ‘**’ 0.01 ‘*’ 0.05 ‘.’ 0.1 ‘ ’ 1  MDRTB = multidrug resistant tuberculosis | | | | | | | |
